# Long-Term Outcomes of Radial Head Arthroplasty in Complex Elbow Fracture Dislocation

**DOI:** 10.3390/jcm10163488

**Published:** 2021-08-07

**Authors:** Alvin Chao-Yu Chen, You-Hung Cheng, Chih-Hao Chiu, Chun-Ying Cheng, Yi-Sheng Chan

**Affiliations:** 1 Bone and Joint Research Center, Department of Orthopaedic Surgery, Chang Gung Memorial Hospital-Linkou, Taoyuan 333, Taiwan; ckbaboon@gmail.com (Y.-H.C.); joechiu0115@gmail.com (C.-H.C.); orthhand@adm.cgmh.org.tw (C.-Y.C.); chan512@cgmh.com.tw (Y.-S.C.); 2 College of Medicine, Chang Gung University, Taoyuan 333, Taiwan

**Keywords:** radial head arthroplasty, elbow fracture dislocation, monopolar prosthesis, radiolucency

## Abstract

The purpose of the current study was to investigate the long-term outcomes of radial head arthroplasty in complex elbow injuries through radiographic analysis and functional correlation. We evaluated 24 radial head arthroplasties in 24 consecutive patients with complex elbow fracture dislocation. All patients were treated with a single type of modular monopolar prosthesis containing smooth stem in press-fit implantation. Clinical survey using the Mayo Elbow Performance Score (MEPS), self-reported scales of shortened Disabilities of the Arm, Shoulder, and Hand (QuickDASH) and the visual analog scale (VAS) at more than 10-year follow-up were reported and compared to 2-year outcomes. Periprosthetic osteolysis was measured in the 10 zones of prosthesis-cortical interface with a modified radiolucency score, which was calibrated by each prosthesis size. Pearson correlation analysis was performed to detect the association between periprosthetic radiolucency and clinical assessment. At the final follow-up, MEPS, QuickDASH score and VAS score averaged 82.5 ± 15, 14.1 ± 14.3 and 1.6 ± 1.2 respectively. A decline in functional status was noted, with decreased mean MEPS and increased mean QuickDASH and VAS scores as compared to the 2-year results while the difference was insignificant. Periprosthetic osteolysis was more prevalent around stem tip of zone 3 and zone 8. The final and 2-year radiolucency scores averaged 7.4 ± 4.2 and 2.6 ± 2.3 respectively with significant difference. Pearson correlation analysis indicated that the difference between radiolucency scores and clinical outcomes in MEPS/QuickDASH/VAS was −0.836, 0.517 and 0.464. Progression of periprosthetic osteolysis after postoperative 10 years is more prevalent around the stem tip with moderate to high correlation to clinical outcomes. Sustained follow-up is warranted to justify subsequent surgery for revision or implant removal.

## 1. Introduction

Complex fracture dislocation of the elbow, most commonly termed terrible triad, involves radial head fracture, coronoid fracture and elbow dislocation with various degrees of ligamentous injuries. Late sequelae with improper management could be complicated with residual instability, development of traumatic arthrosis and joint stiffness [1]. Since the first introduction by Speed in 1941 [2], radial head prosthesis have been widely adopted to replace the irreparable radial head and to reestablish a stable elbow joint allowing ligamentous healing [3,4]. With evolution of implant design and technical refinement, short to mid-term functional results in radial head arthroplasty (RHA) are good to excellent. However, complication rates in the literature vary widely, with reoperation rates ranging from 0 to 45% [5]. Late complications of RHA in terrible triad injuries including painful loosening, osteolysis, capitellar erosion and progressive ulnohumeral arthrosis are commonly described [6,7]. However, most articles have inadequate follow-up and underestimate the failure rate [8]. Long-term outcome following RHA has yet to be further investigated. The aim of this study was therefore to evaluate the long-term outcomes of primary RHA through objective functional survey and self-reported questionnaires in patients of elbow fracture dislocation at a single institute. Complications and radiographic analyses at more than 10 years after surgery were reported and hypothesized to be correlated with objective functional outcomes.

## 2. Materials and Methods

### 2.1. Study Design and Patients

**Figure 1:**
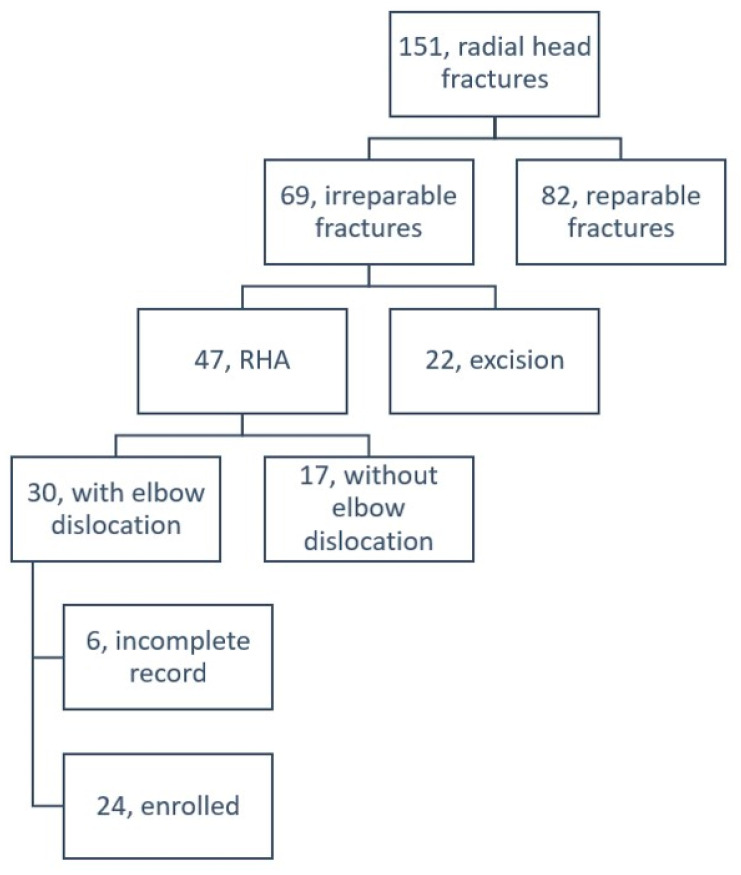
Patient number and surgical profiles in radial head arthroplasty (RHA).

Between May 2004 and March 2011, surgical interventions were performed consecutively in 151 radial head fractures in our institute (Figure 1). Among those fractures, 69 were irreparable fractures and categorized as Mason types III and IV; simple excisions were performed in 22 and RHA, 47 fractures, respectively. All the surgeries were approved preoperatively by the audit committee with surgical indication well documented in the medical records. 

Based on retrospective chart review, 30 out of the 47 fractures in an equal number of patients receiving RHA exhibited concomitant elbow dislocation and were reviewed for this retrospective study. Finally, 24 patients with last follow-up at more than 10 years were enrolled while the other six patients including five loss follow-ups and one deceased were excluded. All were unilaterally injured, consisting of 17 with terrible triad injury, three with Monteggia fracture and three with transolecranon fracture dislocation and one with concomitant distal humerus fracture. There were 18 male and six female patients with a mean age of 42.7 ± 13.3 years (ranging from 24 to 75). Right elbow injury was involved in 14 patients with 10 on the dominant side. Left elbow injury was involved in 10 patients with three on the dominant side. Time from injury to RHA averaged 2.1 ± 3.9 months (ranging from 0 to 16 months). RHA was performed as a primary surgery in 15 patients and as a second procedure in nine patients owing to failed fixation surgery.

### 2.2. Operation

All surgeries were performed under general anesthesia and in supine position with the injured arm supported on a hand table. In 21 patients, radial head fracture was explored with lateral Kocher approach, and three patients were treated by a posterolateral Boyd approach. Open reduction and plate fixation was performed for three patients with concomitant olecranon fractures to restore the original ulnar length. Then, the radial head fragments were removed with proper preparation of the radial neck and medullary canal for prosthesis implantation. RHA was performed with an uncemented modular prosthesis (EVOVLE radial head system, Wright Medical Group, Arlington, TN, USA) consisted of a head segment and a smooth stem. The size of the head segment was 1 to 2-mm downsized after the assembling of radial head fragments in a sized tray. The stem diameter was decided by sequentially reaming the canal of proximal radius. The final height of implanted prosthesis was determined by a combination of proper head thickness and neck length. Intraoperative mini-c-arm fluoroscopy was used to examine the implant position with the proximal margin to reach or 1mm beyond the horizontal level of coronoid tip.

In all 24 patients, lateral ligament-capsular structure was torn and reattached by either Mitek GII anchor (Mitek Surgical Products, Norwood, MA, USA) or Twinfix Ti anchor (Smith & Nephew Endoscopy, Andover, MA, USA) with suture augmentation using No.2 ethibond (Ethicon, Somerville, NJ, USA) following completion of radial head replacement. Medial collateral ligament was explored and fixed with suture anchor repair in patients who present grade III or more instability on valgus test after radial head prosthesis implantation.

### 2.3. Postoperative Care

Long arm splint was applied postoperatively with forearm in neutral rotation at 90° of elbow flexion and sling immobilization for six weeks. Active flexion and extension exercises were started at six weeks after surgery. Forearm pronation and supination exercises were performed actively with the elbow in 90° of flexion while forearm pronation with shoulder abduction was yet prohibited for another 4 weeks. An extension night splint was employed for elbows that were stable in extension to optimize restoration of terminal elbow extension. After eight weeks, active and passive stretching and strengthening exercises were initiated.

### 2.4. Clinical Evaluations

The study protocol was approved by Institutional Review Board (IRB 201800206B0) for patient data retrieval and clinical evaluation. Implant data of radial head prostheses were obtained from the National Health Insurance Administration Register, which contained original registration files and claim records for reimbursement. Collection of functional data was performed by one of the co-authors, who was blinded to the patients’ demographic files. Functional survey was performed using the Mayo Elbow Performance Score (MEPS) and self-reported scales of shortened Disabilities of the Arm, Shoulder, and Hand (QuickDASH) score. Grading on residual pain by visual analog scale (VAS) ranged from 0 to 10.

### 2.5. Radiographic Investigation

**Figure 2:**
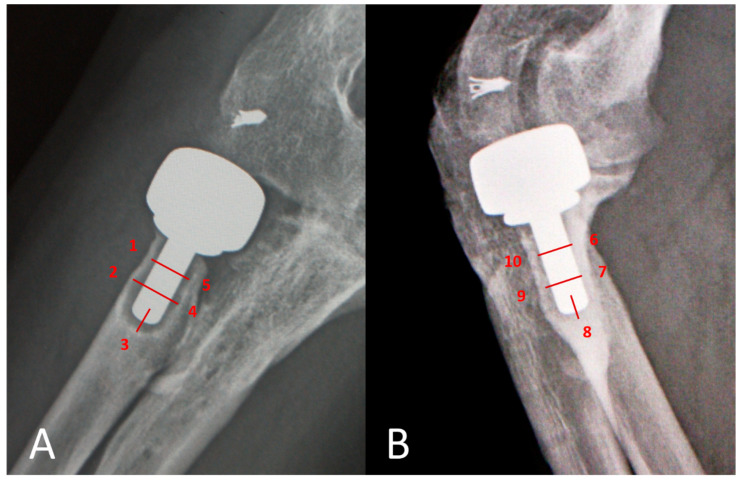
Prosthesis-cortical interface is divided into 5 zones in (**A**) anteroposterior view (zone 1 to 5) and (**B**) lateral view (zone 6 to 10) respectively.

**Figure 3:**
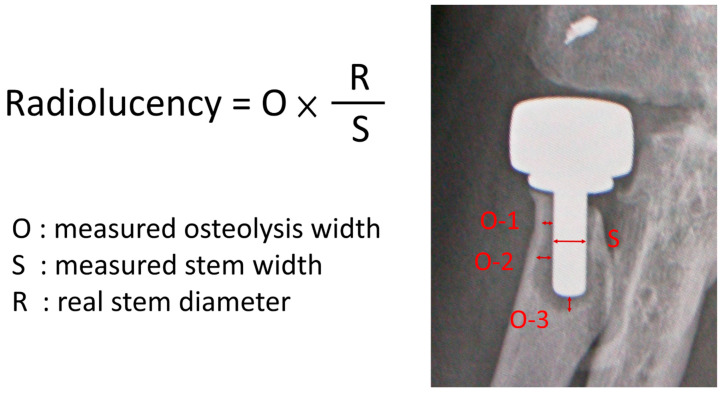
The measured osteolysis width (O) in each of the 10 zones is calibrated with the ratio of the measured stem width on radiograph to the real stem diameter to obtain a real radiolucency thickness.

Anteroposterior (AP) and lateral projections were performed for radiographic evaluation in each elbow. AP view was taken with forearm in full supination and elbow in full extension; lateral view, with elbow in 90° flexion and forearm in neutral rotation. All the images were encrypted to mask the recognizable patient data and reviewed by one single co-author. Radiographic evaluation included radiolucency around the prosthesis stem, presence of osteoarthrosis, and heterotopic ossification (HO). High resolution images of AP and lateral views were meticulously compared with directly postoperative radiographs to identify the location of emerging periprosthetic osteolysis, which was modified according to Gruen method from hip replacement literature [9,10] but only divided into five zones in AP and lateral views respectively (Figure 2). Evaluation of periprosthetic osteolysis on radiograph was performed by drawing a line across the stem at each zone beside the stem and a longitudinal line through the stem tip. The measured width of osteolysis width was then calibrated with stem diameter to obtain a real radiolucency size (Figure 3). A calculated radiolucency thickness greater than 1 mm was considered positive osteolysis at each zone. The sum of radiolucency measurement in all the 10 zones from AP and lateral projections was recorded as radiolucency score of each elbow. All the data in radiographic analyses were rechecked and confirmed by another co-author, who was also blinded to the patient information of the radiographic images.

The degree of degenerative change was graded using the system outlined by the Broberg and Morrey osteoarthritis scale [11] with grade 0 representing a normal joint; grade 1, a slight joint space narrowing and minimum osteophyte formation; grade 2, a moderate joint space narrowing and moderate osteophyte formation; grade 3, a severe degenerative change with gross destruction of the joint. HO was graded based on both location and function restrictions according to the Hastings and Graham classification, and its location and functional restrictions in the range of motion [12].

### 2.6. Statistical Analysis

Statistical analysis was conducted using SPSS version 22.0 (IBM Corp., Armonk, NY, USA). Pearson correlation analysis was applied to identify the correlation between radiolucency and functional outcomes at the follow-up. Wilcoxon signed rank test was used to compare continuous outcomes for two non-normally distributed groups of 2-year and final follow-ups. A *p*-value less than 0.05 was defined as statistically significant.

## 3. Results

All patients returned for follow up at an average of 11.3 ± 1.6 years (range, 10 to 16 years) after RHA. Clinical survey revealed an average MPES of 82.5 ± 15 (range, 45 to 100) with the results of 10 patients rated as excellent, eight good, five fair and one poor. Self-reported outcomes based on QuickDASH questionnaire averaged 14.1 ± 14.3 (range, 0 to 50). Functional results were compared to 2-year data in our previous study [13,14] with an average MEPS of 84.2 ± 14.4 (ranging from 45 to 100) and QuickDASH score of 12.2 ± 12.6 (ranging from 0 to 43.8) respectively (Table 1). There was no significant difference in both MEPS (*p* = 0.442) and QuickDASH scores (*p* = 0.306). The mean VAS score in current study and 2-year data were 1.6 (range, 0–5) and 1.5 (ranging 0–3) respectively; the difference was insignificant (*p* = 0.337). Press-fit implantation was confirmed on all the directly postoperative radiographs that served as a basic control for subsequent radiolucency measurement on 2-year and 10-year radiographs in each patient. Assessment of periprosthetic osteolysis according to radiolucency score was 7.4 ± 4.2 (ranging from 1 to 16.3) from recent radiographs and 2.6 ± 2.3 (ranging from 0 to 6.7), 2-year survey; the difference was significant (*p* = 0.000). None of the 24 patients received subsequent revision RHA or implant removal at latest follow-up and afterwards.

**Figure 4:**
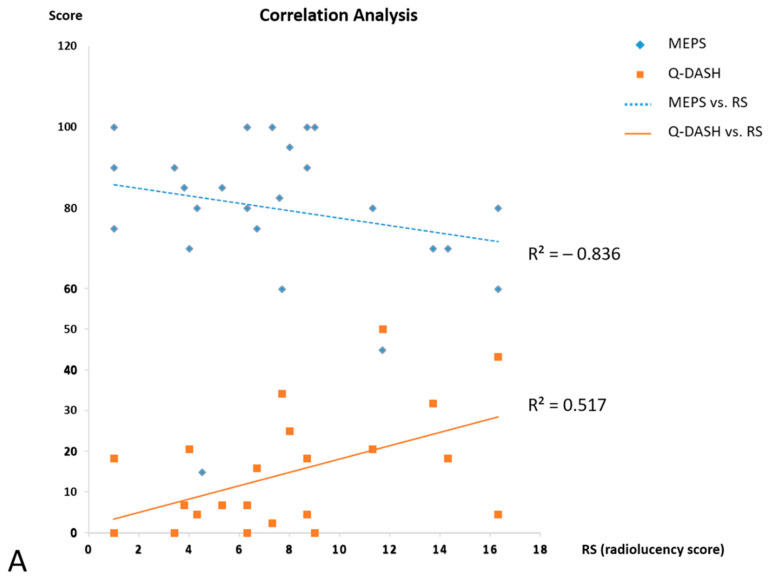

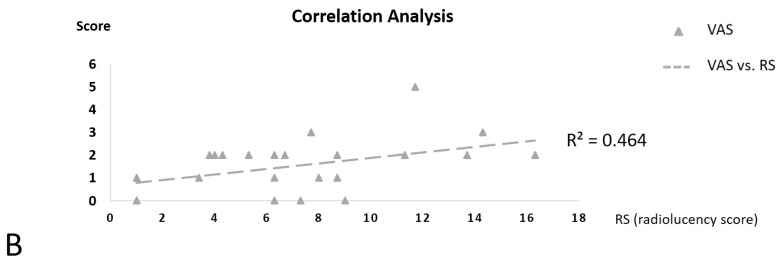
Correlation analysis between radiolucency score (RS) and clinical outcomes. (**A**) High correlation between MEPS, Pearson correlation coefficient R^2^ = −0.836; moderate correlation between RS and Q-DASH, R^2^ = 0.517. (**B**) Moderate correlation between RS and VAS scores, R^2^ = 0.464. MEPS: Mayo Elbow Performance Score; Q-DASH: QuickDASH (shortened Disabilities of the Arm, Shoulder, and Hand); VAS: visual analog scale.

**Figure 5:**
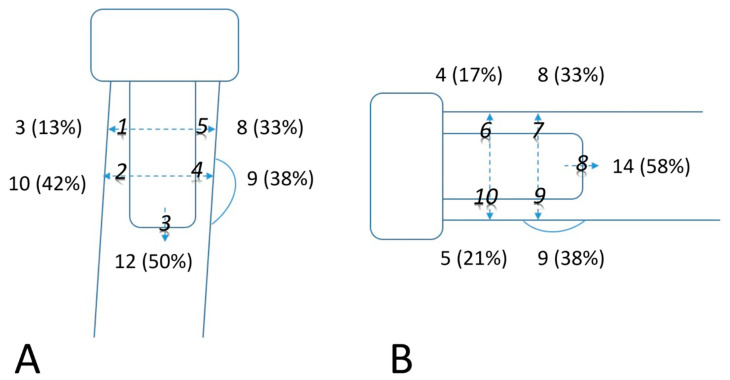
Case number of positive osteolysis defined as ≥1 mm radiolucency thickness in each of 10 zones around implant stem. (**A**) Anteroposterior view. (**B**) Lateral view.

Spearman’s rank-order correlation was used to analyze the association of long-term outcomes and osteolysis measurement. Moderate to high correlation was noted between radiolucency scores and clinical results including MEPS, QuickDASH score and VAS score (Figure 4); Pearson correlation coefficients were −0.836, 0.517 and 0.464, respectively. Radiolucency scores exhibited a negative, high correlation with MEPS, which indicated a detrimental effect in long-term functional status. The distribution of periprosthetic osteolysis in all the 10 zones was showed on Figure 5. Zone 8 on lateral view and zone 3 on AP view exhibited the highest prevalence of osteolysis with positive rates of 58% and 50% respectively, followed by zone 2 (42%) on AP view, and then zone 4 (38%) on AP view and zone 9 (38%) on lateral view. None of the 24 patients were found to have complete radiolucency all around the stem on AP and lateral radiographs, which was defined as prosthesis loosening by Gruen. Other radiographic assessment regarding articular degeneration was analyzed based on Hastings and Graham classification; there were nine patients rated as grade 1, and one patient as grade 2. HO formation was found in nine patients with five in grade 1, three in grade 2-A and one in grade 2-C.

## 4. Discussion

The main strength of this report is the fact it presents a long-term cohort study in elbow fracture dislocation after RHA from a single institute. Clinical investigation was based on both objective functional evaluation and patient self-reported questionnaires at more than 10 years after the index surgery. Radiographic analyses were obtained from high resolution images. Radiolucent distribution around the implant stem was categorized from a modified Gruen method that had been used in the study of monoblock radial head prostheses. Radiolucent thickness was meticulously measured with calibration by the real size of the implanted prosthesis. The major findings included long term changes in functional outcomes, prevalence of radiolucency distribution, and osteolysis progression. Besides, moderate to high association was concluded through Spearman’s correlation analysis between radiographic findings and functional results.

Being an important secondary stabilizer of the elbow, replacement of radial head is advised in case of complex injury with irreparable fracture and ligament tear. Overall, mid- to long-term outcomes are promising in monopolar radial head prosthesis with the longest average follow-up nearing 10 years in the reported publications [4,8,15,16,17]. Osteolysis around the stem has been generally concerned and described in about 50% of all patients with a press-fit radial head prosthesis; prevalence ranged from 17% to 100% in the review of various articles [4]. Given that relatively higher incidence of osteolysis had been reported in complex elbow injuries of terrible triad and Monteggia fractures [18,19], long-term influence of periprosthetic radiolucency has to be further investigated and elucidated.

Periprosthetic osteolysis around the implant stem exhibited significant progression in the current study after more than 10-year follow up. In our observation, osteolysis was most commonly seen around the stem tip of zone 3 and zone 8, followed by area around the distal half of stem, and least commonly seen around the area of radial neck. This finding is due to the smooth stem design of monopolar prosthesis, which is intended to permit greater motion around the stem rather than the space of radiocapitellar contact [20]. Greater motion of stem tip with high prevalence of surrounding radiolucency could be further attributed to the neck-shaft angulation [21] with the resulting discrepancy between the axis of implant stem and that of the forearm rotation.

Controversy still exists regarding adverse effects of periprosthetic osteolysis, which may potentially lead to surgery of revision or implant removal for symptom relief [22]. However, long-term influence on functional status in clinical reports seems inconclusive owing to variable length of follow-up [4]. A mid-term report using smooth-stemmed prosthesis with an average of 8-year follow-up concluded no functional deterioration and no correlation of radiolucency in mid-term outcomes [15] while a recent publication proclaimed radiolucency thickness of 2 mm or more may exhibit positive association with clinical symptoms [23]. Given that considerable variability commonly existed among radiolucency studies owing to different methodology and prosthetic design [24], all the measured thickness of radiolucency and diameter of prosthetic head component was obtained from high resolution electronic images with calibration to the real prosthesis size to minimize measurement variability. Progression of periprosthetic osteolysis was confirmed with significant difference between 2-year and last follow-ups. Other radiographic findings included lower grade articular degeneration and HO formation, which was comparable to other reports in the systemic review [4]. There was a decline in functional status through MEPS, QuickDASH and VAS surveys at final follow-up while not significantly different from the 2-year results. In reviewing three articles with long-term follow-up in modular monoblock prostheses, clinical outcomes were variable; presence of periprosthetic osteolysis was not associated with the change of functional scores [15,25,26]. However, none of those articles measured the thickness and investigated the progression of osteolysis around the implant stem. In our study, there was negative, high correlation between radiolucency score and MEPS, which could imply a detrimental influence of periprosthetic osteolysis in long-term outcomes and prosthesis longevity. Correlation analysis revealed moderate to high correlation between radiolucency score and deteriorated functional status, which was contrary to the common conclusions from mid and long-term reports in the literature. This finding would caution the need for extended observation following RHA and may justify surgical concerns for subsequent revision or removal of loose implants.

Limitations of this study include the retrospective design, the low patient number with lack of adjustment for gender/age/comorbidity, the multiple surgeons involved, and the possible type II statistical error due to the small sample size. In addition, this study also included conversion cases after failed fixation that might exhibit inferior surgical outcomes. Lastly, the excluded data in the cases with insufficient follow-up may exert possible influence on the statistical results.

## 5. Conclusions

Long-term outcome of RHA using modular smooth-stemmed prosthesis is promising after 10-year follow-up in our study. Periprosthetic osteolysis is present in all patients, and is more commonly observed around stem tip. Moderate to high correlation of radiolucency score with clinical outcomes including pain and functional assessment warrants sustained and meticulous investigation. Further prospective trials are needed to verify our findings and the benefit of removal surgery can just be elucidated with long-term studies.

## Figures and Tables

**Table 1:** Comparison of clinical outcomes and radiolucency scores.

Outcome Survey	10-Year Results	2-Year Results	*p*-Value
MEPS	82.5 ± 15	84.2 ± 14.4	0.348
QuickDASH score	14.1 ± 14.3	12.2 ± 12.6	0.306
VAS score	1.6 ± 1.2	1.5 ± 0.8	0.337
Radiolucency score	7.4 ± 4.2	2.6 ± 2.3	0.000 *

MEPS: Mayo Elbow Performance Score; QuickDASH score: shortened Disabilities of the Arm, Shoulder, and Hand score; VAS: visual analog scale. * A *p*-value of <0.05 indicated significant difference.

## Data Availability

The datasets generated during the current study are kept in the databank of Chang Gung Bone and Joint Research Center and are available from the corresponding author on reasonable request.
